# Occupational Risk of COPD: Insights from a Large Cohort Study

**DOI:** 10.1016/j.shaw.2025.08.003

**Published:** 2025-08-23

**Authors:** Haoxuan Yu, Zhiping Yu

**Affiliations:** 1 Department of Civil Engineering, School of Engineering, Monash University Malaysia, Selangor, Malaysia; 2 Monash Climate-Resilient Infrastructure Research Hub (M-CRInfra), School of Engineering, Monash University Malaysia, Selangor, Malaysia; 3 Director of Gastroenterology, Nanzheng District People's Hospital, Hanzhong, China

**Keywords:** economic stability, gender disparities, labor inequality, occupational health, worker health protection

## Abstract

Chronic obstructive pulmonary disease (COPD) remains a critical public health challenge globally. While tobacco smoking is the most recognized risk factor, occupational exposures—especially to vapors, gases, dusts, and fumes (VGDF)—are increasingly acknowledged as substantial contributors. This study offers a secondary reanalysis of publicly available Canadian data, originally collected through the occupational disease surveillance system (ODSS), to investigate COPD risk across diverse occupational sectors and gender strata. By transforming the original hazard ratio data into intuitive visual representations—including scatter plots, histograms, violin plots, and bar charts—we expose previously overlooked gender disparities and risk clusters. Notably, female workers in cleaning, textile, and food preparation services face equally high or even elevated risks compared to men in construction or manufacturing. Our findings underscore methodological limitations in prior studies—such as insufficient detail in indirect smoking adjustment, reliance on outdated occupational coding systems, and lack of individual-level variables—and emphasize the need for gender-sensitive surveillance, policy-oriented communication, and international data-sharing frameworks. This study reframes occupational COPD not only as a biomedical condition but as a social justice issue shaped by labor inequality and surveillance blind spots.

## 1 Introduction

Chronic obstructive pulmonary disease (COPD) is a progressive and debilitating respiratory condition that poses a substantial burden on global health systems [1]. Characterized by persistent airflow limitation, chronic bronchitis, and emphysema, COPD is not only one of the leading causes of death worldwide but also a major contributor to years lived with disability [2]. While tobacco smoking has been widely acknowledged as the most prevalent cause of COPD [3], it is increasingly recognized that occupational exposures—especially to vapors, gases, dusts, and fumes (VGDF)—constitute a significant yet underappreciated etiological pathway for disease development [4]. According to the joint World Health Organization-International Labour Organization (WHO–ILO) global monitoring report [5,6], workplace exposures to particulate matter and chemical irritants are responsible for a substantial proportion of work-related deaths and disability-adjusted life years (DALYs) linked to respiratory diseases.

In this context, occupational COPD has emerged as a distinct clinical and public health challenge. Numerous studies have identified increased COPD incidence and mortality among workers in high-risk sectors, including construction, mining, agriculture, transportation, and manufacturing [7]. Despite growing epidemiological evidence, policy-level attention to occupational health in relation to chronic respiratory conditions remains inadequate, particularly in low- and middle-income countries, where regulatory frameworks often lag behind industrial expansion.

A recent large-scale cohort study conducted in Ontario, Canada (Sritharan et al., *Scientific Reports*, 2024) [8] provided a uniquely comprehensive dataset by examining over 1.5 million workers across multiple industries and identifying 44,138 incident COPD cases. This study offered detailed sex-stratified hazard ratios (HRs) for a wide array of occupations, revealing consistent patterns of elevated COPD risk among workers exposed to VGDF. Significantly, it also highlighted pronounced gender disparities in occupational exposure, with female workers in food preparation, textile processing, and cleaning services exhibiting disproportionately high HRs.

Given the richness and granularity of these published findings, there is an opportunity to further interpret and reframe the results through a gender- and policy-oriented lens. The present analysis undertakes a structured re-evaluation of the published hazard ratio data, with the aim of:

• Visually reconstructing occupational COPD risk across sectors and sexes;
• Highlighting patterns of gendered vulnerability;
• Contextualizing these risks within the broader discourse of global occupational health disparities and sustainable development.

Rather than replicating the original statistical analyses, this work seeks to synthesize, visualize, and interpret the implications of these findings for policymaking, particularly as they pertain to worker protections, transnational regulatory gaps, and gender equity in occupational safety standards.

## 2 Methodology

This analysis is a secondary evaluation and visualization of hazard ratio data extracted from the study conducted by Sritharan et al. (2024) [8], which examined the incidence of COPD among Ontario workers using the occupational disease surveillance system (ODSS) [9]. The ODSS is a longitudinal, population-scale surveillance initiative that links accepted lost-time claims from the Ontario Workplace Safety and Insurance Board (WSIB) to individual health administrative records. These include physician billing data (OHIP), hospital discharges (Discharge Abstract Database, DAD), and emergency/ambulatory care records (National Ambulatory Care Reporting System, NACRS).

Workers were eligible for inclusion if they had a non-COPD work-related injury claim between 1983 and 2019 and were aged 35–65 years at the time of claim [10]. Occupational information was coded using the 1971 Canadian Classification Dictionary of Occupations (CCDO), allowing classification at three levels: division (broad), major, and minor [11]. COPD cases were identified based on administrative health records, with at least three COPD-related OHIP or NACRS entries within two years, or a single qualifying hospital record (ICD-9: 491–492, 496; ICD-10: J41–J44). The final analytical cohort included 1,565,259 workers and 44,138 incident cases (29,445 males; 14,693 females).

A key methodological challenge in occupational health research is accounting for smoking—a dominant risk factor for COPD. Since individual smoking data were not available in ODSS, the original authors applied an indirect adjustment method. This involved deriving smoking prevalence estimates from the Canadian Community Health Survey (CCHS) using eight pooled cycles (2007–2014) [12], stratified by sex, age group, and North American Industry Classification System (NAICS) code. These prevalence rates were then crosswalked with the Standard Industrial Classification (SIC) codes used in ODSS. The adjusted HRs were reported for each occupation group, both before and after controlling for smoking prevalence.

In this secondary analysis, no individual-level data were accessed. Instead, we extracted hazard ratio values and confidence intervals from the published tables, figures, and supplementary materials. These values were then reorganized and visualized through four key graphics:

Scatter plot mapping sex-specific HRs across occupations to identify clustering of high-risk jobs.

• Histogram showing the distribution of HRs by sex to detect skewness and overall risk patterns;
• Violin plot illustrating the density and spread of HRs for male and female workers, highlighting gender-specific exposure disparities;
• Bar chart with error bars comparing high-risk industries and their 95% confidence intervals for precision interpretation.

No further statistical modeling was undertaken. The goal of the visualizations is to aid interpretation of the published findings, with particular attention to gendered occupational risk structures, policy blind spots, and the broader implications for workplace health equity.

To ensure transparency and reproducibility, this reanalysis was conducted entirely using publicly available data published under a Creative Commons Attribution (CC-BY 4.0) license. All hazard ratio values and confidence intervals were extracted from published figures and supplementary tables; no individual-level health records, employment histories, or personally identifiable information were accessed or utilized.

Importantly, the intent of this secondary analysis is not to replicate or challenge the original statistical modeling undertaken by Sritharan et al., but rather to reinterpret and reframe their findings for enhanced scientific communication and policy relevance. By presenting the same epidemiological data through gender-stratified visualizations, this study supports translational policymaking and facilitates evidence-based advocacy on occupational health equity.

## 3 Results

The reconstructed analysis confirms strong and consistent associations between occupational exposure and COPD risk across multiple industrial sectors. The visualizations collectively support three key findings: (1) COPD risk is elevated across a wide range of occupations involving airborne exposures; (2) gender disparities are evident and concentrated in specific sectors; and (3) these disparities persist even after accounting for smoking, implying that occupational factors contribute independently to disease burden.

### 3.1 High-risk sectors and pollutant exposure

**Fig. 1:**
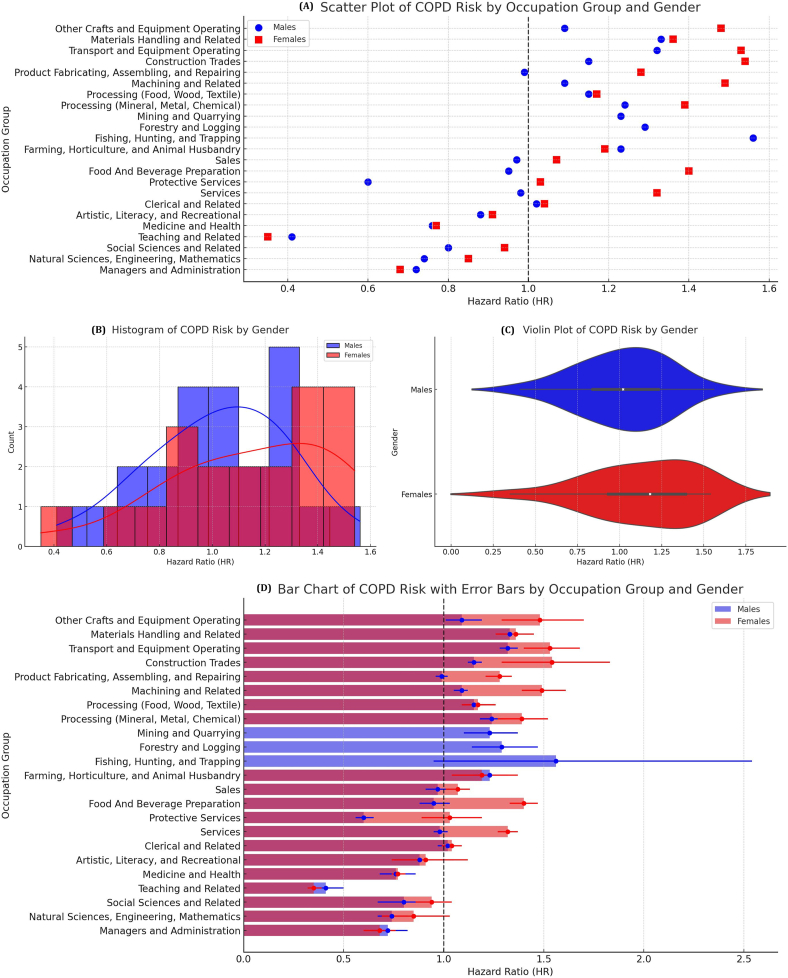
Occupational risk of COPD by gender and occupation group. (A) Scatter plot of COPD risk by occupation group and gender. (B) Histogram of COPD risk by gender. (C) Violin plot of COPD risk by gender. (D) Bar chart of COPD risk with error bars by occupation group and gender. COPD, chronic obstructive pulmonary disease.

As shown in Fig. 1A, occupations such as construction (HR male: 1.15, HR female: 1.54), transportation (HR male: 1.32, HR female: 1.53), and materials handling (HR male: 1.33, HR female: 1.36) cluster in the upper right quadrant of the scatter plot, indicating elevated HRs for both men and women. Additional high-risk occupations include mining (HR male: 1.23) and processing (HR male: 1.24, HR female: 1.39), consistent with known exposures to mineral dust, diesel exhaust, and chemical fumes. In contrast, managerial and administrative roles exhibit reduced risks (HR male: 0.72, HR female: 0.68), serving as a reference for low-exposure occupational environments.

### 3.2 Distributional patterns of occupational risk

The histogram (Fig. 1B) reveals a right-skewed distribution of HRs across both sexes. The modal HR cluster lies between 1.0 and 1.2, indicating that most occupations confer at least some elevated risk compared to the general worker population. The right tail is more prominent among female-dominated occupations, with multiple roles exceeding HR 1.4.

### 3.3 Gender-specific vulnerabilities

In Fig. 1C, the violin plot illustrates that while male workers experience a broader spread of HRs across occupations, certain female-dominated sectors exhibit concentrated high risks. For example, women in food and beverage preparation and cleaning services both report HRs of 1.40, while female workers in textile processing reach HR 1.14. These results suggest that women in low-wage, low-status occupations may experience high exposure to respiratory hazards, often without sufficient protective equipment or regulatory oversight. This is consistent with ILO reports indicating that over 80% of domestic workers globally lack occupational health protections.

### 3.4 Sectoral comparison with precision estimates

Fig. 1D, the bar chart with error bars, offers a comparative look at sex-stratified HRs for selected industries. Transportation, mining, and manufacturing stand out as consistently high-risk sectors, with overlapping confidence intervals indicating robust and statistically significant trends. These results corroborate the widespread underestimation of COPD risk in industries often assumed to be adequately regulated.

Importantly, the smoking-adjusted estimates from the original study suggest that occupational exposures contribute to COPD risk independently of personal smoking behavior. This underlines the need for occupational health interventions that go beyond smoking cessation campaigns and address workplace air quality, protective equipment, and job-specific hazard mitigation.

## 4 Discussion

### 4.1 Reframing COPD risk through gender and policy lenses

This study extends the findings of Sritharan et al. (2024) [8] by conducting a comprehensive secondary analysis with a strong emphasis on gender-sensitive interpretation and policy relevance. While the original study identified significant associations between occupational categories and COPD incidence using HRs, it did not elaborate extensively on how these respiratory health risks manifest differently across gendered labor divisions or visualize the complex epidemiological data in a manner that could effectively inform regulatory frameworks or targeted workplace health interventions.

In contrast, our analytical approach deliberately restructured the published HR values into intuitive and accessible visual forms—including detailed scatterplots, frequency histograms, violin plots, and comparative bar charts—to systematically uncover latent trends, risk clusters, and health disparities that may be systematically overlooked in traditional tabular presentations. This comprehensive visual methodology enabled the clear identification of concerning risk concentrations in traditionally feminized labor sectors such as commercial cleaning services, textile manufacturing, food preparation and service industries, and healthcare support roles, where women appear to face equal or substantially greater respiratory health hazards compared to men employed in conventionally recognized high-risk sectors such as construction, heavy manufacturing, or precision machining.

Our analysis reveals a troubling pattern of occupational health invisibility that disproportionately affects women workers. The traditional focus on male-dominated industrial sectors has created systematic blind spots in occupational health surveillance, regulatory frameworks, and prevention strategies. Female-dominated occupations often involve chronic exposure to cleaning chemicals, textile dust, biological aerosols, and fine particulate matter—exposures that may be less dramatic than those found in heavy industry but are nonetheless significant contributors to the respiratory disease burden. Furthermore, the intersection of gender, class, and occupational risk creates compound vulnerabilities. Many women in high-risk occupations are employed in precarious work arrangements with limited access to comprehensive health benefits, occupational health services, or meaningful workplace safety protections.

The findings fundamentally suggest that occupational COPD should not be understood merely as an isolated epidemiological phenomenon, but rather as a complex social and gendered health issue that reflects systematic historical neglect in occupational safety standards, regulatory oversight, and health protection mechanisms for informal, service-oriented, and care work roles that have been traditionally undervalued in labor market hierarchies.

### 4.2 Revisiting the original study's limitations and methodological considerations

While Sritharan et al. (2024) [8] made a substantial and valuable contribution to occupational health research by analyzing COPD risk patterns using a large-scale linked administrative dataset spanning multiple decades, several important methodological constraints and analytical limitations in their original study restrict its broader interpretability and practical application—issues that our comprehensive reanalysis seeks to highlight, contextualize, and address through a critical methodological lens.

First, the original study employed what they described as an “indirect adjustment” methodology for smoking behavior using population-level data from the CCHS, but critically did not provide clear specification of the precise statistical approach utilized—such as whether a standardized mortality ratio (SMR), standardized incidence ratio (SIR), or a more sophisticated regression-based adjustment method was implemented. This methodological opacity significantly limits the reproducibility of the smoking adjustment procedure and may systematically obscure the extent of residual confounding effects, particularly when examining differential risk patterns across gender categories, age cohorts, and specific occupational classifications. To address this limitation, our reanalysis focuses exclusively on the smoking-adjusted HRs already published by the original study and visualizes them through intuitive, sex-stratified graphics. By avoiding further modeling on top of an unclear adjustment process, we enhance interpretability without introducing additional uncertainty, and highlight gendered exposure disparities that remain visible even after smoking adjustment—thus demonstrating the robustness and policy relevance of these patterns.

Second, VGDF (vapors, gases, dusts, and fumes) exposure classification in the original analysis was primarily inferred using broad occupational codes derived from the 1971 CCDO, without subsequent validation through contemporary job-exposure matrices (JEMs), expert industrial hygiene panels, or modern workplace exposure assessments. This methodological reliance on outdated occupational classification systems introduces substantial measurement uncertainty and potential exposure misclassification, particularly for occupational categories that have undergone significant technological evolution, regulatory changes, or industrial transformation since the 1970s. Recognizing this limitation, our study does not attempt to redefine or reclassify occupational codes, but instead visually maps the distribution of risk across occupational groups to identify systematic clusters of elevated HRs—particularly in sectors often neglected in traditional VGDF exposure assessments, such as cleaning, food preparation, and textile work. This approach offers an alternative lens to detect exposure-related disparities that might be masked by outdated coding systems and encourages future efforts to refine exposure classification based on observed risk patterns.

Third, the linked administrative dataset utilized in the original study did not include critical individual-level variables such as detailed medical comorbidities, socioeconomic indicators, employment duration and intensity, personal protective equipment utilization patterns, specific worksite environmental conditions, or workplace safety culture measures. The systematic absence of these important covariates significantly restricts the analytical ability to disentangle occupation-specific respiratory risk from broader social determinants of health, healthcare access patterns, and lifestyle factors that may confound the observed associations [13,14]. To partially mitigate these data limitations, our reanalysis prioritizes gender-stratified visualizations that reveal persistent disparities across occupational groups even after adjustment for smoking. While we acknowledge the inability to control for all unmeasured confounders, this visual approach enables the identification of structural patterns—such as the concentration of high risk in low-wage, female-dominated sectors—that suggest the presence of systemic vulnerabilities unlikely to be explained solely by missing covariates. In doing so, we offer a complementary interpretive framework that broadens the scope of inference beyond statistical control and emphasizes real-world exposure inequities.

Additionally, the potential for occupational misclassification, diagnostic uncertainty in COPD identification, and temporal changes in occupational coding systems were not addressed comprehensively in the original analysis. These methodological limitations are particularly concerning when attempting to generate precise risk estimates for policy development or targeted intervention design. To address this challenge, our approach emphasizes visual distributional analysis rather than precise point estimation. By employing comparative graphics with confidence intervals—such as bar charts and scatter plots—we convey the relative magnitude and spread of risk across sectors, allowing readers to detect robust patterns even in the presence of classification noise or diagnostic ambiguity. This strategy facilitates more intuitive interpretation for nontechnical audiences and supports preliminary prioritization in occupational health policy, particularly in under-protected or overlooked sectors.

Finally, while the original research presented rigorous statistical outputs—such as HRs and confidence intervals—it offered limited exploration of their practical implications, policy relevance, or gendered health equity concerns. In particular, the respiratory risks faced by workers in female-dominated service sectors remained underemphasized, and the numerical presentation format did little to support engagement from policymakers, occupational health professionals, or advocacy groups seeking actionable insights. In contrast, our reanalysis translates complex quantitative findings into visually intuitive, gender-stratified formats that reveal hidden patterns and disparities. By embedding these insights within broader social and policy contexts—including labor hierarchies, regulatory blind spots, and healthcare access—we not only enhance the interpretability of occupational COPD risks, but also reframe them as urgent issues of structural inequality and public health justice.

Collectively, this critical reanalysis demonstrates how visual, gender-sensitive, and policy-oriented reinterpretation of existing data can overcome key methodological blind spots in large-scale occupational health studies. Rather than replicating complex modeling, our approach adds value through accessibility, clarity, and contextual nuance—revealing structural inequities that may otherwise remain hidden. These findings highlight the need for a paradigm shift in occupational epidemiology: one that centers lived experience, incorporates intersectional risk patterns, and prioritizes translational insight over statistical abstraction. By making the invisible visible, especially for historically overlooked worker populations, this work lays the foundation for more inclusive and equitable policy development in occupational health.

### 4.3 Implications for policy development and future research directions

Despite the inherent limitations of secondary data analysis, our findings generate several meaningful and actionable implications for both future research priorities and evidence-based policy development initiatives at local, national, and international levels.

First, the clear clustering of elevated HRs in traditionally female-dominated occupational sectors calls for the immediate development and implementation of gender-specific occupational health surveillance systems and targeted intervention strategies. Existing occupational disease registries, workers' compensation systems, and public health monitoring programs often systematically underreport adverse health outcomes among informal workers [15,16], service sector employees, and care workers, particularly in employment contexts where personal protective equipment use is inconsistent and regulatory oversight mechanisms are minimal or inadequately enforced.

Second, the methodological approach demonstrated in this study—systematically repurposing publicly available epidemiological data for policy translation and advocacy applications—can serve as a practical and replicable model for low- and middle-income countries (LMICs) and resource-constrained settings where comprehensive occupational health data infrastructure remains limited or underdeveloped. The Ontario Disease Surveillance System (ODSS) represents a global best-practice example of integrated administrative data linkage for population health surveillance, but broader international collaboration, technical assistance, and capacity-building initiatives are urgently needed to adapt and implement similar surveillance models across different national contexts, labor market structures, and health system configurations.

Third, significant methodological innovations are urgently needed to address current limitations in occupational health research and practice. Priority areas include the development of updated, gender-sensitive job-exposure matrices that account for modern work arrangements, informal employment conditions, emerging chemical and biological hazards, and contemporary industrial practices and safety technologies [17,18]. Advanced approaches, such as linking traditional occupational surveillance data with emerging environmental sensor networks, wearable health monitoring devices, electronic health record systems, and real-time workplace exposure monitoring, can dramatically enhance both the detection of emerging respiratory hazards and the implementation of evidence-based prevention strategies [19,20]. Furthermore, the integration of machine learning approaches, natural language processing of occupational health records, and sophisticated spatial analysis techniques offers promising opportunities to identify previously unrecognized exposure-disease associations and develop more precise, personalized prevention recommendations [21,22].

Future research initiatives should systematically incorporate robust qualitative research components designed to understand and document the lived experiences, workplace challenges, and health impacts experienced by workers with COPD, particularly among women, immigrant communities, and other marginalized populations who may face additional barriers to healthcare access and workplace safety advocacy. Community-based participatory research approaches that center worker knowledge, experiences, and priorities can also help identify practical intervention strategies, policy recommendations, and advocacy approaches that are more likely to be effective, sustainable, and responsive to community needs and priorities.

### 4.4 Conclusion and future directions

Through systematic gender-stratified data visualization, critical policy analysis, and comprehensive methodological review, this study offers a unique and valuable reinterpretation of complex COPD risk patterns embedded within contemporary occupational structures and labor market dynamics. By highlighting previously under-examined occupational sectors, demonstrating the practical utility of data visualization for public health translation and advocacy, and providing a critical analysis of existing methodological approaches, this research contributes to a more inclusive, equitable, and comprehensive model of occupational health research and practice.

While the inherent limitations of secondary data analysis, exposure misclassification, and unmeasured confounding remain important considerations that cannot be fully resolved through reanalysis alone, the clarity, accessibility, and policy relevance of the insights presented strongly support the value and utility of this study as both a methodological case example and a compelling empirical narrative of systematic health inequity, occupational respiratory exposure, and public health system neglect that warrants urgent attention, substantial resource allocation, and coordinated action across multiple sectors and levels of governance.

Moving forward, the integration of advanced analytical methods, community-engaged research approaches, and policy-relevant data visualization represents a promising pathway toward more effective, equitable, and sustainable occupational health protection for all workers, regardless of gender, employment sector, or socioeconomic status.

## CRediT authorship contribution statement

**Haoxuan Yu:** Writing – review & editing, Writing – original draft, Visualization, Validation, Supervision, Project administration, Data curation, Conceptualization. **Zhiping Yu:** Writing – review & editing, Writing – original draft, Supervision, Project administration.

## Ethical statement

Not applicable.

## Statement on the use of AI tools

During the preparation of this work, the authors did not use generative AI or AI-assisted technologies to generate any part of the content. All sections of the manuscript, including the writing, analysis, figures, and revisions, were completed solely by the authors without the assistance of AI tools. The authors take full responsibility for the integrity and originality of the work.

## Funding

H.Y. thanks the Graduate Research Excellence Scholarship (GRES) from Monash University Malaysia.

## Conflict of interest

The authors declare that they have no known competing financial interests or personal relationships that could have appeared to influence the work reported in this paper.
